# Controlling Nonlinear Dynamics of Milling Bodies in Mechanochemical Devices Driven by Pendular Forcing

**DOI:** 10.3389/fchem.2022.915217

**Published:** 2022-08-05

**Authors:** A. Polo, M. Carta, F. Delogu, M. Rustici, M. A. Budroni

**Affiliations:** ^1^ Dipartimento di Chimica e Farmacia, Università Degli Studi di Sassari, Sassari, Italy; ^2^ Dipartimento di Ingegneria Meccanica, Chimica, e Dei Materiali, Università Degli Studi di Cagliari, Cagliari, Italy

**Keywords:** transition to chaos, forced-damped oscillators, modeling of mechanochemical systems, mechanochemistry, ball-mill grinding

## Abstract

Understanding the dynamics of milling bodies is key to optimize the mixing and the transfer of mechanical energy in mechanochemical processing. In this work, we present a comparative study of mechanochemical reactors driven by harmonic pendular forcing and characterized by different geometries of the lateral borders. We show that the shape of the reactor bases, either flat or curved, along with the size of the milling body and the elasticity of the collisions, represents relevant parameters that govern the dynamical regimes within the system and can control the transition from periodic to chaotic behaviors. We single out possible criteria to preserve target dynamical scenarios when the size of the milling body is changed, by adapting the relative extent of the spatial domain. This allows us to modulate the average energy of the collisions while maintaining the same dynamics and paves the way for a unifying framework to control the dynamical response in different experimental conditions. We finally explore the dynamical and energetic impact of an increasingly asymmetric mechanical force.

## 1 Introduction

Mechanochemistry is emerging with increasing strength as a powerful approach to the synthesis of fine chemical compounds (Friscic, 2012; James et al., 2012; Balaz et al., 2013; Boldyreva, 2013; Wang, 2013; Rightmire and Hanusa, 2016; Do and Friščić, 2017; Tan and García, 2019; Friscic et al., 2020; Porcheddu et al., 2020). In contrast with conventional chemistry in solution, which makes use of heat and light to activate and drive the chemical reaction, mechanochemical transformations are caused by the application of mechanical forces to solid phases (Thiessen et al., 1967; Butyagin, 1971; Thiessen, 1979; Boldyrev, 1983; Avvakumov, 1986; Heinicke, 1986; Michalchuck et al., 2020).

In light of the common use of manual and mechanical grinding throughout human history, and starting from the XIX century in particular (Takacs, 2013), the mechanical activation of chemistry does not represent a novelty in itself. However, the possibility to carry out chemical reactions under solventless conditions, or with a significant reduction of solvent phase, is extremely appealing now that attention to green chemistry issues is unavoidable and impossible to procrastinate (Galant et al., 2022). The evidence that mechanical processing can also open synthetic routes to chemicals and materials that cannot be prepared by more conventional methods further explains why mechanochemistry is currently experiencing vigorous growth (Gomollon-Bel, 2019;^1^).

Amongst the mechanical processing methods, ball milling is a popular choice. Contrary to the mechanochemistry of individual molecules, which requires extremely refined manipulation methods (Boldyreva, 2013), ball milling is an easy method for mechanical processing of granular materials (Thiessen et al., 1967; Tan and García, 2019; Friscic et al., 2020; Porcheddu et al., 2020). Widely available in a variety of sizes and designs, ball milling devices are equipped with one or more reactors that contain one or more balls. The balls collide with each other and with the reactor walls as the vessel is shaken in mono-axial, pendular or rotational modes, crushing and deforming at relatively high strain rates the powder particles trapped between the colliding surfaces (Tan and García, 2019; Friscic et al., 2020). The local non-hydrostatic mechanical stresses generated during the impact can finally result in the activation of chemical reactions depending on the intensity of mechanical forces and the physical and chemical properties of the processed material (Thiessen et al., 1967; Tan and García, 2019; Friscic et al., 2020; Porcheddu et al., 2020).

Despite the interest attracted by mechanical processing in the past 50 years, the understanding of how the mechanical energy translates into chemical reactivity is still unsatisfactory. Accordingly, new theoretical tools to understand how to design, control, and predict the development of these processes are needed (Carta et al., 2020; Carta et al., 2021).

As the activation and the progress of a given mechanochemical transformation crucially depend on the conditions experienced by powder particles at collisions and by their effective mixing, one informative approach relies on the study of the dynamics of the milling bodies responsible for dragging the powder and driving collisions.

In the past, various attempts have been made in this direction for the most widespread ball milling devices (Burgio et al., 1991; Abdellaoui and Gaffet, 1995; Courtney, 1996; Magini et al., 1996; Feng et al., 2004; Cleary et al., 2006; Sinnott et al., 2006; Boschetto et al., 2013; Broseghini et al., 2016; Zhao and Shaw, 2017). The dynamics of milling balls in the SPEX Mixer/Mill 8,000 device have also been studied in detail within a deterministic framework (Watanabe et al., 1995; Delogu et al., 1998; Concas et al., 2006). In agreement with experimental results, numerical simulations showed how balls’ trajectories and collisions at the reactor walls are sensitive to the collision elasticity degree (Rustici et al., 1996; Delogu et al., 1998; Delogu et al., 2000), which can control the transition from periodic to chaotic behaviors in the temporal displacement of the ball (Caravati et al., 1999; Manai et al., 2002). From the spatial viewpoint, these chaotic regimes showed a fingerprinting multifractal topology in the location and recurrence of collisions on the reactor walls (Budroni et al., 2014; Budroni et al., 2017).

The onset of chaotic behaviors can greatly favor an effective mixing of the granular medium inside the medium (King, 1998; Aref et al., 2017), and it is, indeed, useful for isolating parametric conditions where these regimes can occur. In this context, we propose a comparative study and classification of mill body dynamics inside reactors driven by harmonic pendular motion, which represents one class of commonly used mechanochemical devices. We focus on two different geometries of reactors characterized either by flat or curved lateral borders (the latter system has never been explored in this perspective). Moreover, two possible designs of the reactors are considered, either with the mechanical arm driving the periodic motion allocated at the center of the reactor (symmetric forcing) or at different distances from it (asymmetric forcing). Symmetric and asymmetric forcing can induce different transfer and dissipation of the energy within the reactor as well as impact spatio-temporal dynamics of the milling body, which has never been deepened. We first explore the onset of dynamical scenarios of the two geometries driven by a symmetric forcing in response to the variation of the collision elasticity and the milling body size. The latter critically impacts mechanochemical processing and is mostly chosen in experiments by following empirical methods and trial-error procedures, hence without any theoretical reference. On the basis of these first results, we check the existence of a guiding criterion to predict the typology of dynamical regimes on the basis of the relative size of the reactor and the milling body. This represents a contribution on the way towards a unifying parametrization of mechanochemical devices, which could help the reproducibility and the scaling-up of the results of mechanical processing, which are carried out in a plethora of different experimental conditions (Gil-González et al., 2021). We finally investigate systematically the effect of an asymmetric forcing on the milling body dynamics.

## 2 Mechanochemical Devices Driven by Pendular Forcing: Models

**FIGURE 1:**
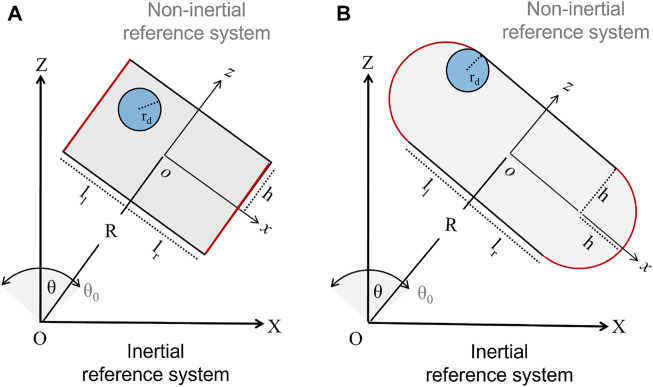
Sketches of two 2-dimensional ball-milling devices driven by harmonic pendular motion (*X*, *Z*) and (*x*, *z*) represent the inertial and non-inertial reference frames, respectively. Panel **(A)** illustrates a reactor with flat bases (in red) of width *l*_*f*_ = *l*_*l*_ + *l*_*r*_ and height 2*h*, sustained by a mechanical arm of length *R*. *l*_*l*_ and *l*_*r*_ regulate the distance of the arm from the reactor center, and when the left and right sides of the reactor are different, *l*_*l*_ ≠ *l*_*r*_, the system experiences an asymmetric forcing. The mechanical treatment is performed by a rigid disc of radius *r*_*d*_. The oscillatory motion is described by the angle *θ*, with maximal amplitude *θ*_0_ = *π*/12, and frequency *ν* = 18 Hz. Panel **(B)** shows a reactor characterized by curved bases of curvature radius *h*, width *l*_*c*_ = *l*_*l*_ + *l*_*r*_ + 2*h* and height 2*h*.

We analyzed systems where the mechanical forcing is transferred to a milling body via a harmonic pendular motion of the reactor. Since in a previous paper we verified that the main dynamical features of these milling body dynamics are preserved in a 2-dimensional description, we focused our attention on this reduced framework. We considered the most common geometries for these classes of devices, as illustrated schematically in Figure 1. The first presents a rectangular shape with flat bases, defining a spatial domain *Ω*_*f*_ = *w*_*f*_ × 2*h*, where *w*_*f*_ and *h* are the reactor width and semi-height, respectively. The other geometry is characterized by semicircular lateral borders (bases) of radius *h*, such that the related spatial domain writes *Ω*_*c*_ = *l*_*c*_ × 2*h* + *πh* ^2^. The milling disc has a radius *r*_*d*_. The reactor is driven by the displacement of a mechanical arm of length *R*, which can be mounted at a variable distance from the reactor center as controlled by parameters *l*_*l*_ and *l*_*r*_, giving the right and left border distance from the reactor center, respectively. In the flat-base model, the system width is thus *w*_*f*_ = *l*_*l*_ + *l*_*r,*_ while *w*_*c*_ = *l*_*l*_ + *l*_*r*_ + 2*h* holds for the curved-base case (i.e., *l*_*c*_ = *l*_*l*_ + *l*_*f*_). When *l*_*l*_ ≠ *l*_*r*_ the milling disc experiences an asymmetric force.

The periodic angular motion of the reactor on the vertical plane follows *θ* = *θ*_0_ cos (*ωt*), where *θ*_0_ is the maximal angular amplitude and *ω* = 2*πν* gives the oscillation frequency. Two Cartesian reference frames can be used to reconstruct the reactor motion and the disc dynamics: the inertial system of coordinates (*X*, *Z*), which is centered at the lower end of the mechanical arm, and the non-inertial reference of coordinates (*x*, *z*), whose origin coincides with the geometrical center of the reactor, where the *x* axis is orthogonal to the mechanical arm. This non-inertial reference frame undergoes rigid displacement with the reactor.

The motion of each point in the system can be described and transformed from the non-inertial to the inertial coordinate system through the following systems of equations:

$$X=x\;\cos\theta +\left(z+R\right)\sin\theta ,$$ (1)

$$Z=-x\;\sin\theta +\left(z+R\right)\cos\theta$$ (2)

and

x=Xcos⁡θ+Zsin⁡θ, (3)

z=Xsin⁡θ+Zcos⁡θ−R. (4)

In the absence of external forces, the disc follows a rectilinear motion started by the impact with a reactor wall.

$$X^{b}\left(t+ht\right)=X^{b}\left(t\right)+V_{x}^{b}\left(t\right)\;ht,$$ (5)

$$Z^{b}\left(t+ht\right)=Z^{b}\left(t\right)+V_{z}^{b}\left(t\right)\;ht,$$ (6)

where $V^{b}\left(t\right)=\left(V_{x}^{b}\left(t\right),V_{z}^{b}\left(t\right)\right)$ is the disc velocity in the inertial reference frame at time *t*, which can be obtained by differentiation of Eqs 1 and 2, and *ht* is the integration time step. The integration is performed by using the Euler algorithm. At each step, the inertial reference frame coordinates of the disc (*X*_*b*_(*t*), *Z*_*b*_(*t*)) are updated by applying Eq. 5, and, from that, the disc coordinates in the non-inertial reference frame (*x*_*b*_(*t*) *z*_*b*_(*t*)) can also be calculated.

Whenever **x**_**b**_ = (*x*_*b*_, *z*_*b*_)∉Ω (i.e., the distance of the disc from the reactor walls is smaller than *r*_*d*_), a collision occurs and the non-inertial component of the disc velocity normal to the impacted surface is instantaneously reversed, modulated by the restitution coefficient *f*. *f* ranges between 0 and 1, giving the inelastic and elastic limits, respectively. This mimics the effect of the powder undergoing a transformation inside the mechanochemical device on the collision efficiency. The term “collision” here identifies any event where the milling disc is at the reactor walls, including slipping phenomena.

As a whole, the dynamics of the milling body describes a forced-damped oscillator, where the impacts with the periodically moving reactor and friction at the walls feature the forcing and the damping contributions to the disc dynamics, respectively. In a sense, our problem features a complicated variant of the classical bouncing ball system (Tufillaro and Albano, 1986).

The duration of any given collision is limited to a single time step. Coherently with this aspect, the integration time step is set smaller than 10^–5^ s (we used *ht* = 10^–7^ s), which is also consistent with experimental data (Delogu et al., 1998) and the Hertzian theory of impacts (Love, 1944; Timoshenko and Goodier, 1970).

It is finally worth noticing that the acceleration experienced by the disc with the oscillation frequency at use is one order of magnitude larger than the gravitational acceleration, and this, together with the shortness of the disc’s mean free path, allows us to consider negligible the gravitational contribution to the disc motion.

## 3 Results and Discussion

### 3.1 Flat vs. Curved Bases

We first investigate the influence of the different shapes of the reactor lateral borders (either flat or curved) on the disc dynamics. To make the comparison between the two geometries as homogeneous as possible, we fixed the reactors’ height to 2*h* and adapted their width in order to get the same spatial domain *Ω*_*f*_ = *Ω*_*c*_. As a reference, we assumed the values of typical devices with semicircular bases (*w*_*c*_ + 2*h* = 57 mm and *h* = 9.4 mm), which are smaller than common Spex-mill apparatuses characterized by flat borders and already characterized in previous work (Caravati et al., 1999; Manai et al., 2002; Budroni et al., 2014). Equivalent areas of the spatial domains *Ω*_*f*_ and *Ω*_*c*_ are thus obtained via the relation *w*_*f*_ = (2*l*_*c*_ + *πh*)/2.

To facilitate the comparison of the two systems, we classified the possible behaviors in the parameter spaces spanning the restitution coefficient, *f*, and the disc radius, *r*_*d*_. From previous studies and preliminary simulations, these parameters remained the most relevant from the dynamical viewpoint, and a numerical benchmark on the effect of these parameters could be useful for practical purposes. The other parameters governing the mechanics of the system were fixed to typical values for these devices *θ*_0_ = *π*/12, *ν* = 18.6 Hz, *R* = 0.122 m. Simulations were run for 50 s and the resulting trajectories were analyzed from a dynamical viewpoint.

To get an integrated dynamical and energetic view, we also evaluated the average number of collisions on the reactor lateral borders (bases), $\left\langle Collisions\right\rangle =N_{b}^{\left(d\right)}/t$ (where $N_{b}^{\left(d\right)}$ is the number of collisions on the bases and *t* the total simulation time) as well as the mean energy, ⟨*E*⟩_*rel*_, averaged over the total number of collisions, $N_{t}^{\left(d\right)}$ .

$$\left\langle E\right\rangle =\frac{m_{d}}{2N_{t}^{\left(d\right)}}\overset{N_{t}^{\left(d\right)}}{\underset{i=1}{\sum }}{\left(v_{i}^{\left(d\right)}\right)}^{2},$$ (7)

where *m*_*d*_ is the disc mass and $v_{i}^{\left(d\right)}$ is the related velocity at the *i*th collision. In our numerical framework, the disc is a material point with an effective size given by *r*_*d,*_ which can influence the dynamics and velocity profile of the disc, while its mass is not explicitly controlled by a parameter. However, we can define the mean collision energy in relation to that of a reference disc with radius *r*_*f*_ (we used *r*_*f*_ = 1 mm) and the same density, *ρ* (such that $m_{d}=\pi \rho r_{d}^{2}$ and $m_{f}=\pi \rho r_{f}^{2}$ ):

$${\left\langle E\right\rangle }_{\mathrm{rel}}=\frac{{\left\langle E\right\rangle }_{d}}{{\left\langle E\right\rangle }_{f}}=\frac{N_{t}^{\left(f\right)}r_{d}^{2}}{N_{t}^{\left(d\right)}r_{f}^{2}}\frac{\sum_{i=1}^{N_{t}}{\left(v_{i}^{\left(d\right)}\right)}^{2}}{\sum_{i=1}^{N_{t}}{\left(v_{i}^{\left(f\right)}\right)}^{2}},$$ (8)

where $N_{t}^{\left(f\right)}$ and $v_{i}^{\left(f\right)}$ are the total number of collisions and the *i*th collision velocity of the reference disc, respectively. Each simulation was started by locating the milling disc in the center of the non-inertial reference system. Changes in the initial position of the disc do not affect the asymptotic dynamical regime unless the system is marginally close to chaotic conditions.

**FIGURE 2:**
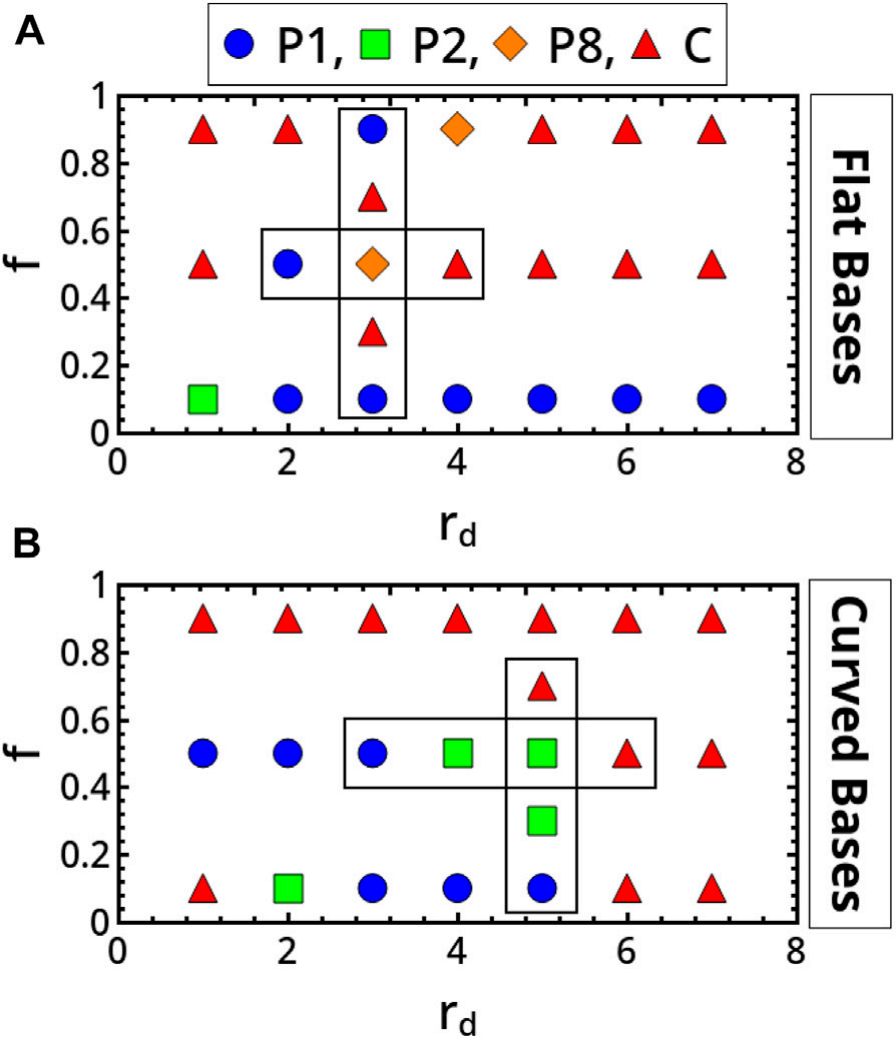
Classification in the parameter spaces spanning the restitution coefficient, *f*, and the disc radius, *r*_*d,*_ of the main scenarios characterizing the milling body dynamics in flat-base **(A)** and curved-base **(B)** geometries. P1, P2, P8 and C identify period-1, period-2, period-8 and chaotic regimes, respectively.

Figure 2 gives a global overview of the typical scenarios pertaining to the two systems, which clearly differ both in qualitative and quantitative terms.

**FIGURE 3:**
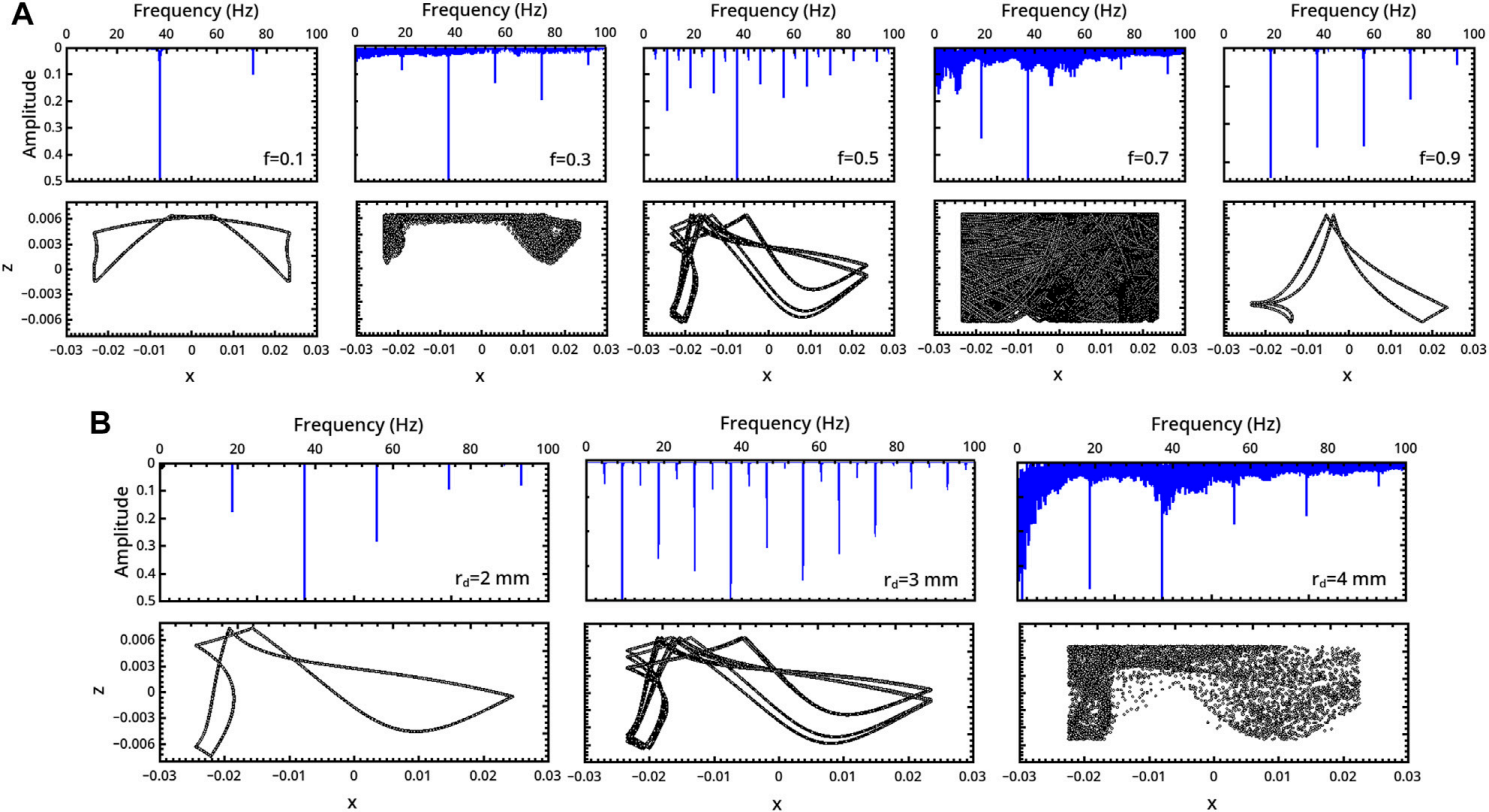
Characterization of the milling body dynamics in a flat-base geometry as a function of **(A)** the restitution coefficient *f* (*r*_*d*_ = 3 mm) and **(B)** the milling disc radius *r*_*d*_ (*f* = 0.5). For each regime, we report the trajectory (bottom) and the FFT of the related series describing the vertical displacement of the milling disc in time (top).

Periodic regimes dominate the dynamics of the reactor with flat bases in inelastic conditions (low *f*). For this geometry, transitions from periodic to aperiodic behaviors can be observed by increasing the restitution coefficient (see for example, the back and forth scenario framed in Figure 2A for *r*_*d*_ = 3 mm). This route to chaos (and back to regular periodicity) is illustrated in Figure 3, where we report the trajectories of the disc in the reactor along with the Fast Fourier Transforms (FFTs) of the vertical displacement of the disc in time (^2^). The transition is characterized by the alternation of periodic and aperiodic regimes which occurs in narrow windows of the control parameters (insignificant changes from the experimental viewpoint). Although a period-doubling scenario seems to regulate the transition, it was difficult to refine the whole sequence of bifurcations. Coarsely, by increasing *f* from 0.1 to 0.3, the system switches from a period-1 (P1) behavior (characterized by a main frequency twice the working frequency of the device) to a chaotic regime. The last regime shows the fingerprinting broad-band FFT profile of chaotic scenarios, with the same dominant frequency as the preceding periodic regime. We further analyzed the aperiodic dynamics by calculating the progressive divergence of two disc trajectories starting from infinitesimally distant initial positions, ‖*δ*_0_‖ ∼ 10^–7^ mm, with the formula ‖*δ*(*t*)‖ ≃‖*δ*_0_‖*e* ^*λt*^ for *t* ≫ 1. Although not calculated from the true phase-space, *λ* can give a rough estimation of the maximal Lyapunov exponent of the system (Strogatz, 1994; Kantz and Schreiber, 1997), whose positive values are a signature of chaotic regimes. Approaching chaos, *λ* goes from small negative values to positive values as reported in Figure 5A.

In the inelastic range *f* ∈ (0.1, 0.3), the milling disc spends most of the time in the upper part of the reactor. The related trajectories show a symmetric pattern with respect to the central *x* = 0 axis in the periodic regime, which transmutes into an asymmetric shape in the chaotic one.

The system undergoes a bifurcation to period-8 (P8) dynamics by further increasing *f* to 0.5 (see the frequency-halving in the related FFT), which preludes a new chaotic regime at *f* = 0.7. Elastic conditions (*f* = 0.9) bring the system back to a P1 periodicity compatible with the forcing frequency.

The dynamics of the disc is also very sensitive to the disc radius, and a transition from periodicity to chaos can be observed when *r*_*d*_ is increased from 2 to 4 mm (with *f* = 0.5). Here also a period-doubling scenario can be inferred, though not all progressive regimes were isolated.

The asymmetric patterns drawn by the disc motion within the spatial domain can be reflected with respect to the axis *x* = 0 by reversing the initial direction of the external angular forcing.

**FIGURE 4:**
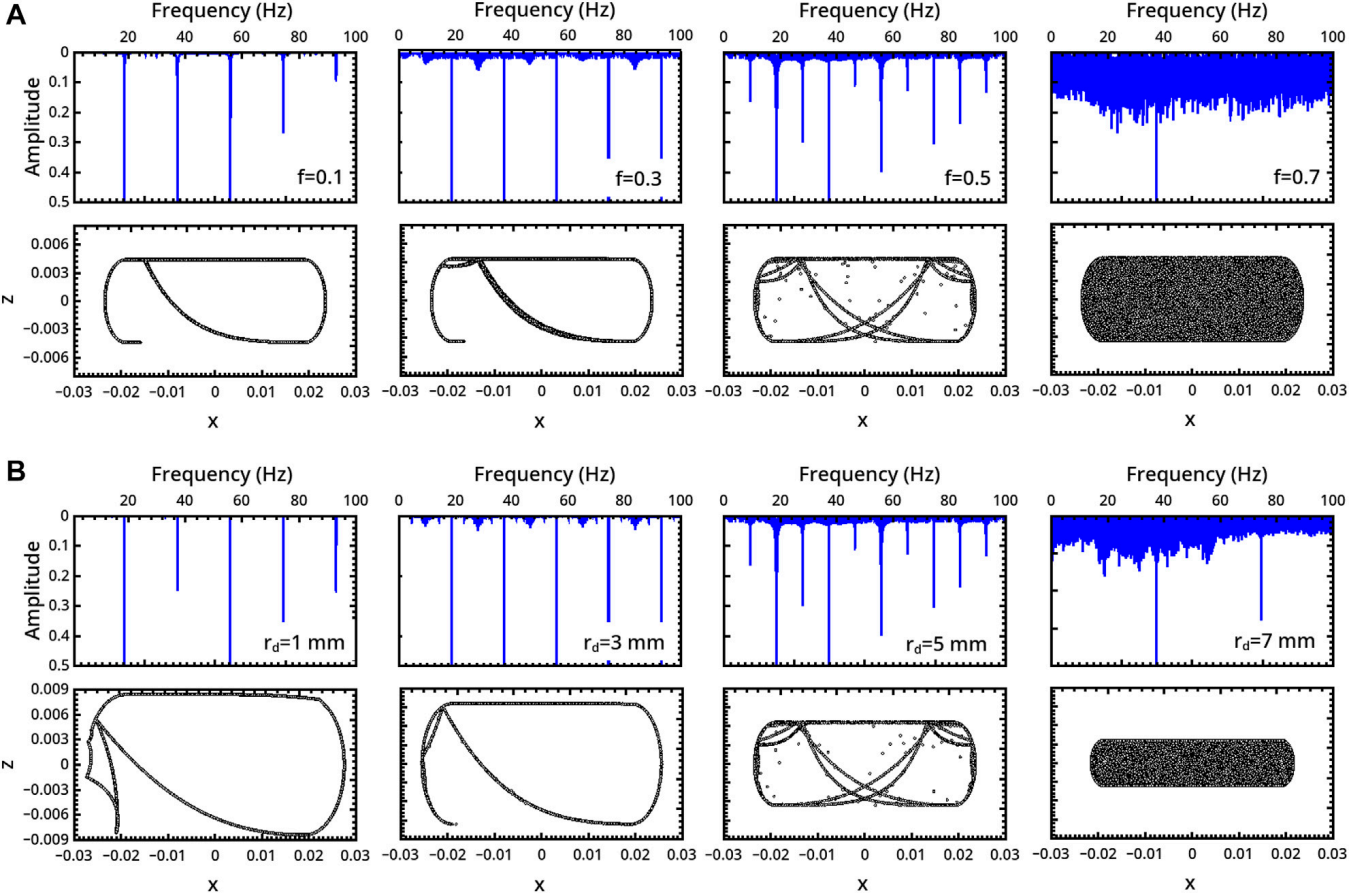
Characterization of the milling body dynamics in a curved-base geometry as a function of **(A)** the restitution coefficient *f* (*r*_*d*_ = 5 mm) and **(B)** the milling disc radius *r*_*d*_ (*f* = 0.5). For each regime, we report the trajectory (bottom) and the FFT of the time series describing the vertical displacement of the milling disc (top).

**FIGURE 5:**
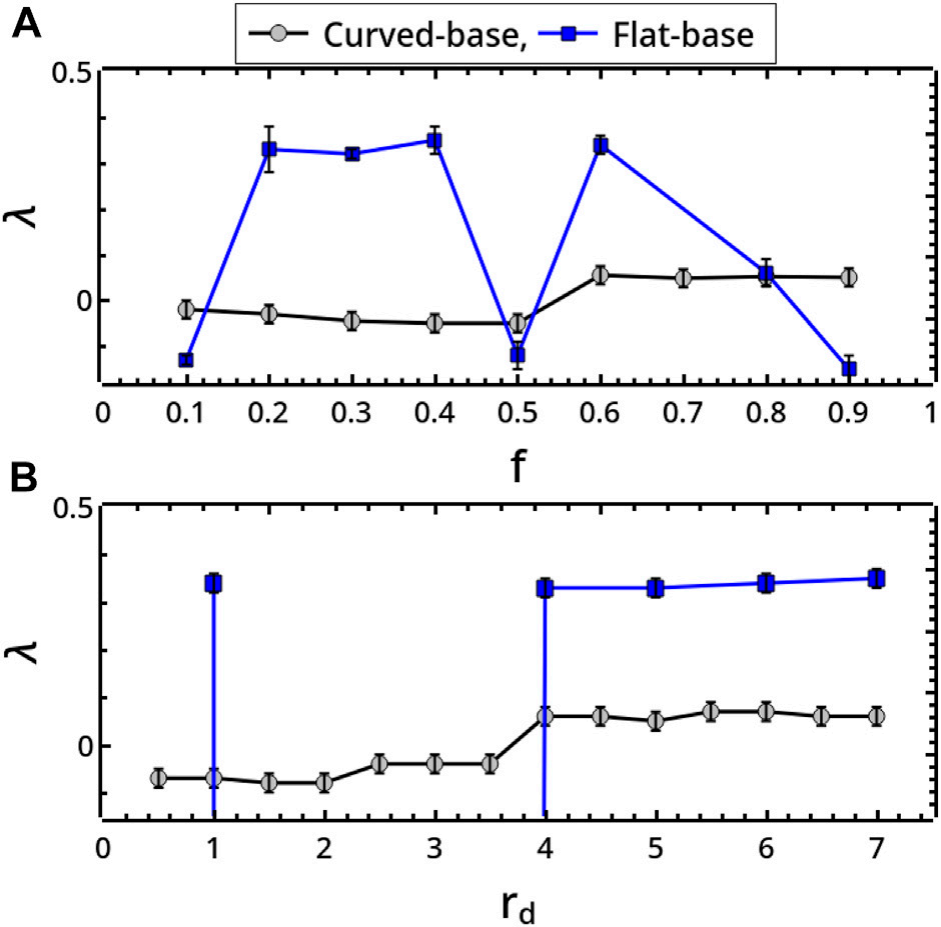
Exponential divergence, *λ*, characterizing milling body trajectories in the flat- and curved-base geometries when changing **(A)** the restitution coefficient *f* (*r*_*d*_ = 5 mm) and **(B)** the milling disc radius *r*_*d*_ (*f* = 0.5). *λ* was obtained through the formula ‖*δ*(*t*)‖ ≃‖*δ*_0_‖*e* ^*λt*^, by calculating the distance ‖*δ*(*t*)‖ at any time *t* between two trajectories starting from infinitesimally distant conditions ‖*δ*_0_‖ → 0 (here ‖*δ*_0_‖ ∼ 10^–7^ mm) for *t* ≫ 1 (here after 50 s) (Kantz and Schreiber, 1997).

In the system with curved bases, aperiodic dynamics are found not only in the elastic domain but also for low values of *f* (see also Figure 2 of the Supplementary Material). Again, the system can evolve from periodicity to chaotic regimes following a period-doubling-like pathway when *f* or *r*_*d*_ are varied as framed in Figure 2B, explicitly illustrated in Figure 4 and characterized by computing the exponent *λ* in Figure 5B.

For both kinds of reactors, periodic regimes correspond to highly ordered trajectories where the milling disc explores a very restricted portion of the available area. However, while in flat-base reactors impacts take place in a few points of the reactor walls, curved-base geometries favor a displacement along the reactor borders dragged by the reactor motion.

**FIGURE 6:**
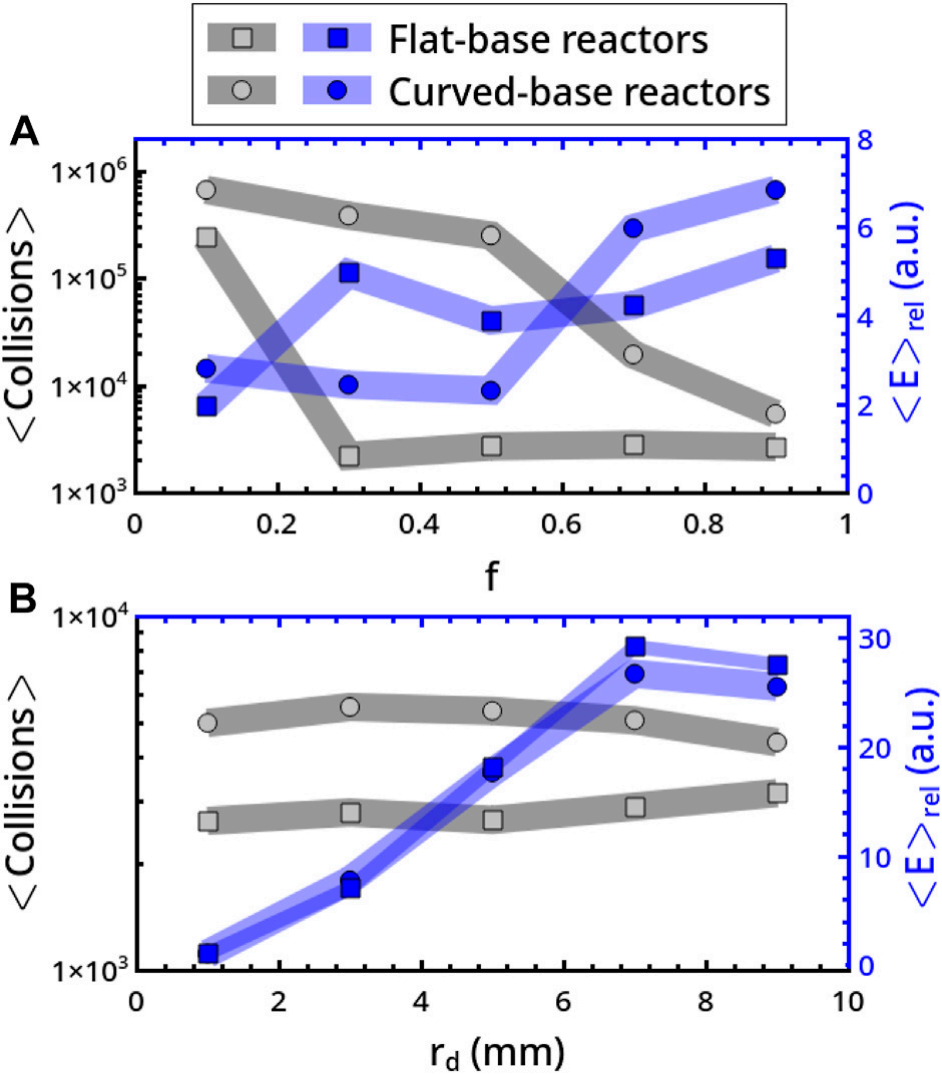
Analysis of the average number of collisions on the reactor bases, ⟨Collisions⟩ (in gray-black), and relative energy per collision, ⟨*E*⟩_*rel*_ (in blue). ⟨*E*⟩_*rel*_ was evaluated in relation to a disc of radius rd = 5 mm, to which we attributed unitary mass. ⟨Collisions⟩ and ⟨*E*⟩_*rel*_ are characterized as a function of *f* (*r*_*d*_ = 3 mm) in panel A and *r*_*d*_ (*f* = 0.9) in panel B. Squares and circles refer to flat- and curved-base reactors, respectively.

This explains why the number of collisions experienced by the disc in the curved-base systems is, in general, much larger than that observed in flat-base reactors. In Figures 6A,B we report the dependence upon *f* and *r*_*d*_ of the average number of collisions on the reactor bases, ⟨Collisions⟩ (in gray-black), and the relative energy, ⟨*E*⟩_*rel*_ (in blue). In flat-base reactors (circles), ⟨Collisions⟩ shows a sharp drop-down as *f* is increased beyond the strongly inelastic condition 0.1 (*r*_*d*_ = 5 mm), while a smoother decreasing trend pertains to curved-base reactors (Figure 6A). For both reactor types, ⟨Collisions⟩ is less sensitive to a variation of *r*_*d*_ and the corresponding trends are essentially constant. The trends for ⟨*E*⟩_*rel*_ of the two geometries are comparable both in qualitative and quantitative terms. The increasing trend with *r*_*d*_ is described by Eq. 8 and is related to the disc mass, which increases correspondingly.

In practical terms, curved-base reactors provide a more efficient mixing, showing wider regions of the parameter space where chaotic dynamics can be accessed and a larger number of collisions, which corresponds to a higher probability of inducing a transformation, but there is a negligible gain in energetic terms as compared to flat-base reactors.

### 3.2 Scaling the Reactor Size

The reactors with different shapes of the lateral borders show divergent dynamics even if the sizes of the milling body and the reactor, as well as all the other mechanical parameters, are the same. However, given the dependence of the dynamics of the two reactors on the size of the milling body, we checked whether systems with the same basis type and the same ratio of the reactor size to that of the milling body converge to analogous regimes. The rationale is that discs with an analogous free space to move, under the same mechanical forcing, could give a similar dynamical response. If this is the case, the dimensionless ratio $Q=\Omega_{f}/\left(\pi {\left(r_{d}\right)}^{2}\right)=\Omega_{c}/\left(\pi {\left(r_{d}\right)}^{2}\right)$ can feature a simplified and unifying parameter to control the dynamics.

We thus explored separately the dynamics of symmetrically forced reactors with either flat or curved bases, keeping fixed *Q*. Specifically, we compared the behaviors of systems where a constant *Q* is obtained by varying the reactor dimensions (*i*) without keeping the reactor aspect ratio (i.e., changing at will the width and the height of the reactor with the length of the mechanical arm, *R*, fixed) (case 1), (*ii*) preserving the aspect ratio of the reactor by scaling its dimensions by the same factor (with *R* fixed) (case 2), and (*iii*) scaling the whole system proportionally, including the length of the mechanical arm (case 3).

**FIGURE 7:**
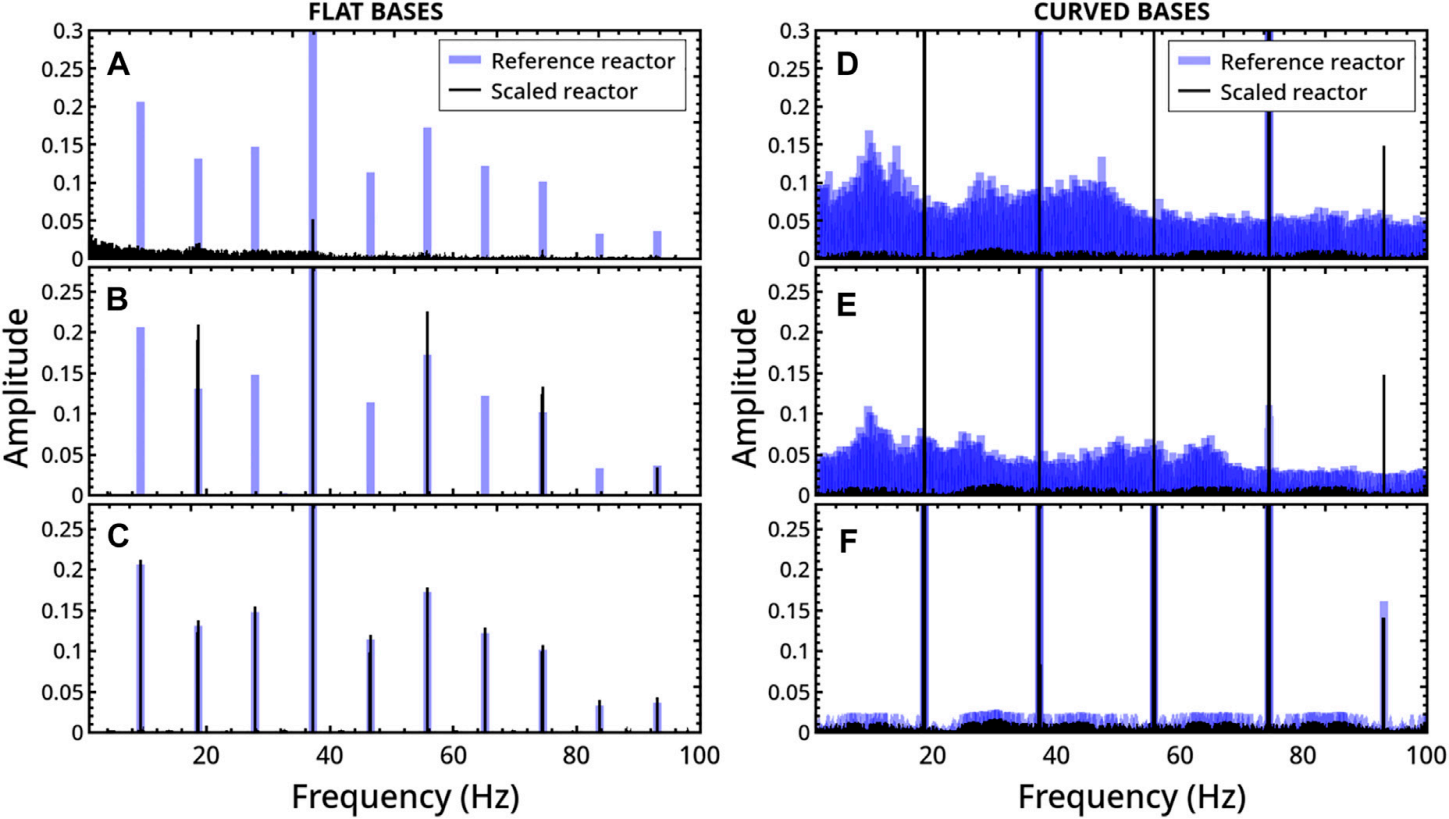
Comparison between the milling disc dynamics obtained by changing the reactor size with the milling disc radius in flat-base (left) and curved-base (right) geometries, keeping fixed the ratio $Q=\Omega_{c}/\left(\pi {\left(r_{d}\right)}^{2}\right)=\Omega_{c}/\left(\pi {\left(r_{d}\right)}^{2}\right)$ (*f* = 0.5). The reference systems, in blue, are characterized by $r_{d}^{1}=3$ mm, $\Omega_{f}^{1}=$ 53 mm × 18.8 mm = $\Omega_{c}^{1}=$ 38.2 mm × 18.8 mm + *π* (9.4 mm)^2^ while the scaled reactors, in black, have $r_{d}^{2}=1.5r_{d}^{1}=$ 4.5 mm, $\Omega_{f}^{2}=\Omega_{c}^{2}=2.25\Omega_{f}^{1}=2.25\Omega_{c}^{1}$ . The comparison between the reference and scaled systems is performed by using the Fast Fourier Transforms of the *z*-displacement of the milling disc over time. The change of the reactor size is operated in three different ways: **(A**–**D)** Case 1: scaling the dimensions without preserving the reactor aspect ratio keeping *R* = 122 mm ( $\Omega_{f}^{2}=$ 53 mm × 42.3 mm; $\Omega_{c}^{2}=$ 104.4 mm × 18.8 mm + *π*(9.4 mm)^2^); **(B**–**E)** case 2: scaling the dimensions by the same factor of the disc radius, preserving the aspect ratio of the reactor and keeping *R* = 122 mm ( $\Omega_{f}^{2}=$ 79.45 mm × 28.2 mm; $\Omega_{c}^{2}=$ 57.3 mm × 28.2 mm + *π*(14.1 mm)^2^); **(C-F)** case 3: scaling the whole system proportionally, including the mechanical arm ( $\Omega_{f}^{2}=$ 78.45 mm × 28.2 mm; $\Omega_{c}^{2}=$ 57.3 mm × 28.2 mm + *π*(14.1 mm)^2^; *R* = 183 mm).

We first comment on the general case 1. Figure 7A shows a representative parallel between the dynamical regimes of two systems with flat bases, one being 2.25 times larger than the other, including the disc area. In particular, there are superimposed the FFTs of the temporal series describing the disc displacement along the vertical axis, *z*. An analogous comparison is reported for reactors with curved bases in Figure 7D (related trajectories can be found in Figure 3 of the Supplementary Material). In both cases, preserving *Q* does not translate into analogous regimes. In the example with flat base reactors, the regular periodicity characterizing the “small” reactor changes into an aperiodic scenario in the scaled reactor. *Viceversa,* a transition from strongly aperiodic to period-1 behavior is observed in the curved-base system. In general, we found that the discrepancy between the dynamics of reference and scaled systems holds from both qualitative and quantitative viewpoints for different combinations of *f* and *r*_*d*_ (becoming much more prominent when approaching elastic conditions, see Figure 2 of the Supplementary Material), and also by increasing the horizontal instead of the vertical dimension.

A similar comparison between systems where the reactor dimensions are scaled proportionally while maintaining the aspect ratio does not indicate any dynamical matching (see an example in Figures 7B,E), unless the mechanical arm is also scaled by the same scale factor, which is shown in Figures 7C,F. In this last case, the trajectory described by the disc within the two scaled reactors follows the same scaled path (see Figure 4 of Supplementary Material) and, as a consequence, the Fourier analysis leads to overlapping results.

**FIGURE 8:**
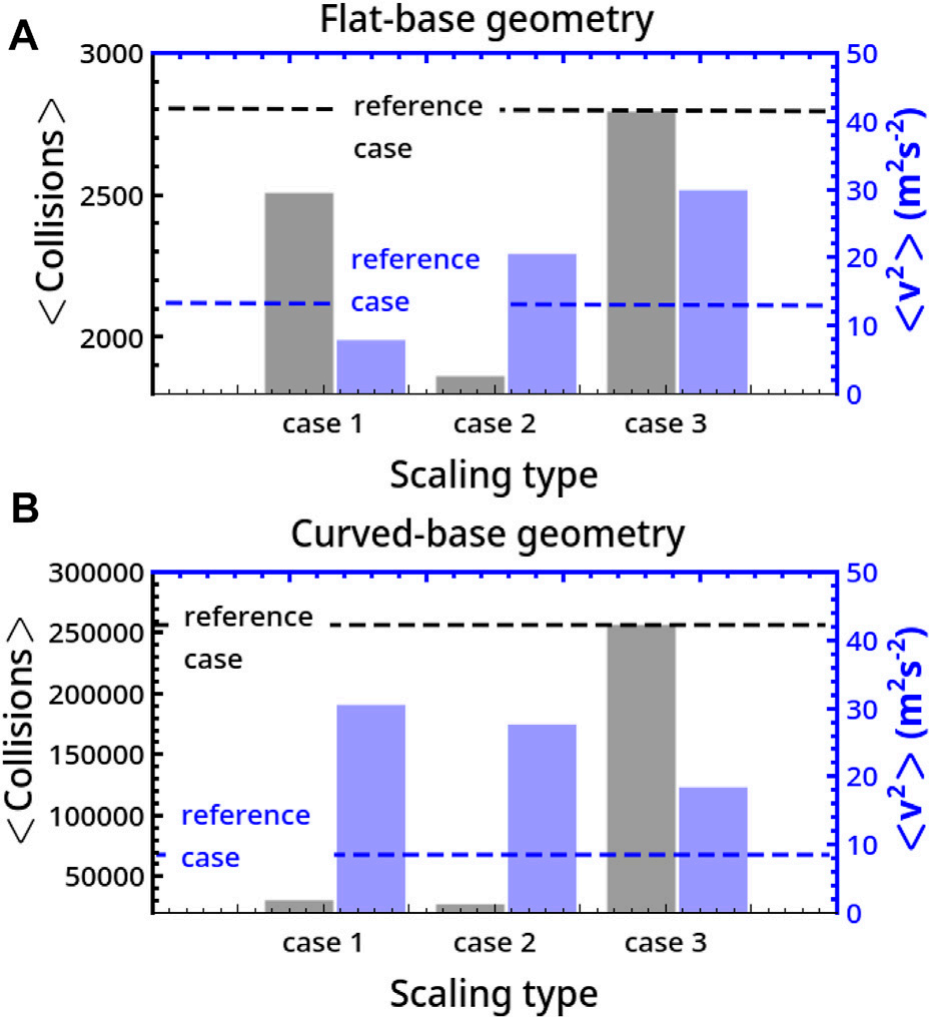
Comparison of the average number of collisions on the basis, ⟨Collisions⟩, and mean squared velocity per collision, ⟨*v* ^2^⟩, for different scaling (cases 1, 2, and 3) of the system size. Panel A and B describe results for flat- and curved-base geometries.

In Figure 8 we can observe that both in flat- and curved-base reactors, the average number of collisions on the bases ⟨Collisions⟩ is maximal for the reference reactor (case 3 coincides with this). By contrast, the average energy per collision will be larger in scaled systems not only because of the increment in the mass of the milling body but also because of an increment in the mean squared velocity of the disc, ⟨*v* ^2^⟩. In particular, in flat reactors, apart from case 1, ⟨*v* ^2^⟩ is larger in the scaled reactors than in the smaller reference system, with ${\left\langle v^{2}\right\rangle }_{\text{case }2}<{\left\langle v^{2}\right\rangle }_{\text{case }3}$ while in curved-base geometries ⟨*v* ^2^⟩ follows the order ${\left\langle v^{2}\right\rangle }_{\text{case }1}>{\left\langle v^{2}\right\rangle }_{\text{case }2}>{\left\langle v^{2}\right\rangle }_{\text{case }3}>{\left\langle v^{2}\right\rangle }_{\text{reference case}}$ .

**FIGURE 9:**
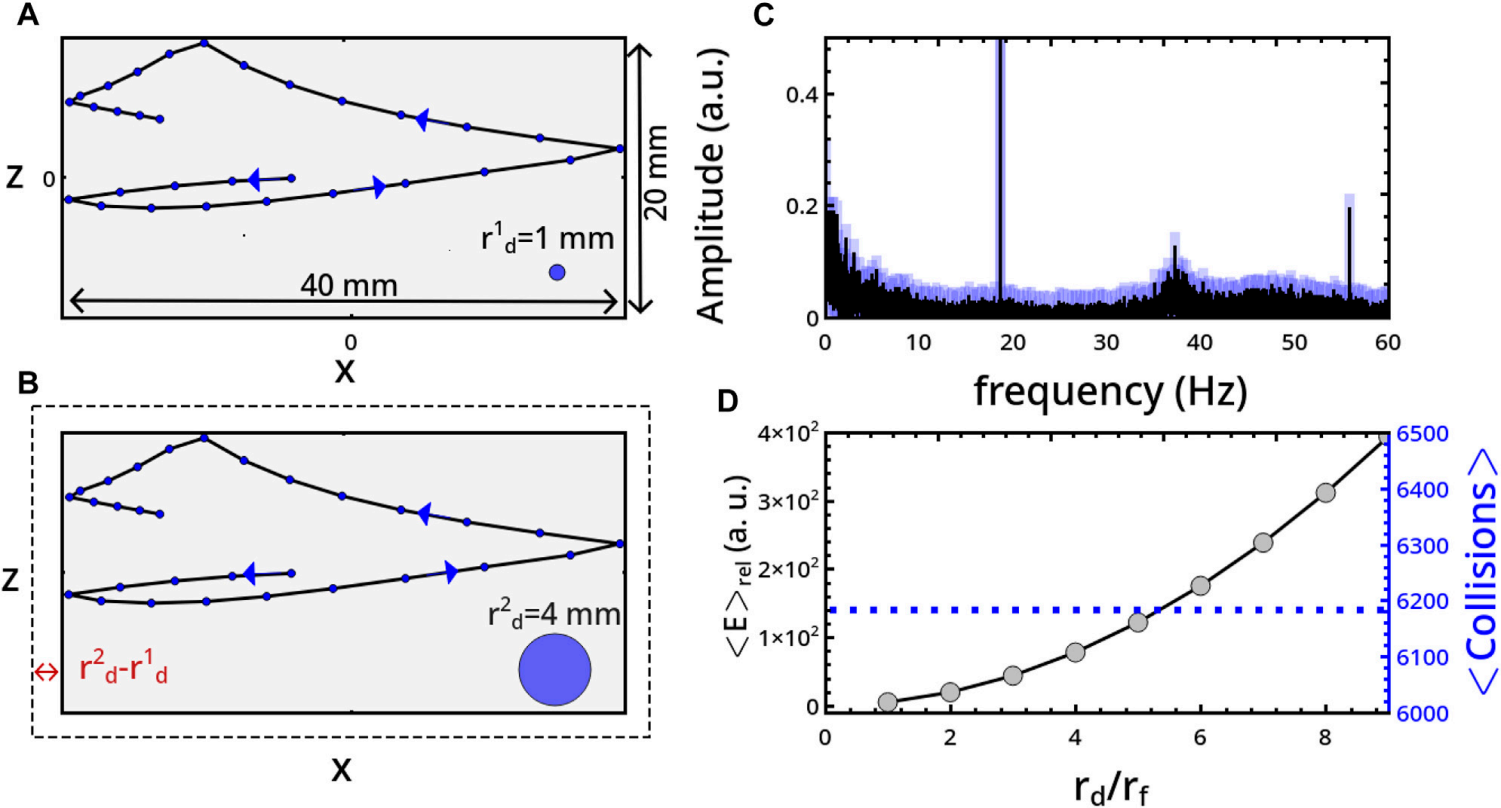
Comparison between the milling disc dynamics obtained with *r*_*d*_ = 1 mm **(A)** and *r*_*d*_ = 4 mm **(B)** (i.e., *ϵ* = 3 mm), by adapting the spatial domain from *Ω*_*f*_ = 20, ×, 40 mm to *Ω*_*f*_ = 26 × 46 mm, according to Eqs 9, 10. *f* is fixed to 0.5. **(C)** Comparison between the FFTs of the *x*-displacement of the milling disc in time. **(D)** Average number of collisions on the bases, ⟨Collisions⟩, and average relative energy per collision, ⟨*E*⟩_*rel*_, with respect to the reference energy of a milling disc with radius *r*_*f*_ = 1 mm.

Another way to relate the dynamics of the milling body to the spatial domain available relies on simple geometrical arguments. Since the conditions for collisions are based on the distance *r*_*d*_ of the disc center from the borders, when *r*_*d*_ increases by, *ϵ*, collision conditions remain unaltered if the dimensions of the reactor are increased by the same quantity *ϵ* at each side in the flat-base and if the reactor half-height increases to *h* + *ϵ* in the curved-base systems. In other words, the dynamics of a disc with a radius $r_{d}^{1}$ will be the same of a milling disc with $r_{d}^{2}$ , if the reactor dimensions change from (*w* ^1^ × 2*h* ^1^) to

$$w^{2}=w^{1}+2\epsilon ,$$ (9)

$$2h^{2}=2h^{1}+2\epsilon ,$$ (10)

$$\left(with\;\epsilon =r_{d}^{2}-r_{d}^{1}\right),,$$

in flat-base geometry, and to

$$h^{2}=h^{1}+2\epsilon ,$$ (11)

in geometries with curved bases. These equivalence criteria, allowing to adapt the system size to a change in the disc radius in order to get the same dynamics, are applied to the representative systems shown in Figure 9. There are reported small portions of the spatial trajectories of a milling disc moving in a flat-base symmetrically-forced reactor when *r*_*d*_ changes from 1 mm (panel (a)) to 4 mm (panel (b)) and, consequently, *Ω*_*f*_ = 20, ×, 40 mm is varied to *Ω*_*f*_ = 26 × 46 mm (*f* = 0.5). The resulting dynamics are identical and show overlapping FFTs of the temporal *x*-displacement in Figure 9C. Analogous results were also found for curved-base reactors (see Figure 5 of the Supplementary Material).

If, in this way, the dynamical regime of the milling body is preserved, things vary from the energetic viewpoint due to the increase of the disc mass. Assuming a constant density of the disc and verified that the velocity distribution does not change while adapting the reactor size, the average energy per collision will only depend upon *r*_*d*_. In particular, ⟨*E*⟩_*rel*_, measuring the average energy per collision of a disc with radius *r*_*d*_ with respect to that of a reference radius *r*_*f*_, follows ${\left\langle E\right\rangle }_{\mathrm{rel}}∼{\left(r_{d}\right)}^{2}/{\left(r_{f}\right)}^{2}$ as shown in Figure 9D, where *r*_*f*_ = 1 mm. Notice how the total number of collisions remains constant while varying *r*_*d*_. This picture introduces a degree of freedom in the control of the system by which a desired regime can be modulated in intensity while maintaining unaltered the dynamical features.

### 3.3 Symmetrical vs. Asymmetrical Forcing

**FIGURE 10:**
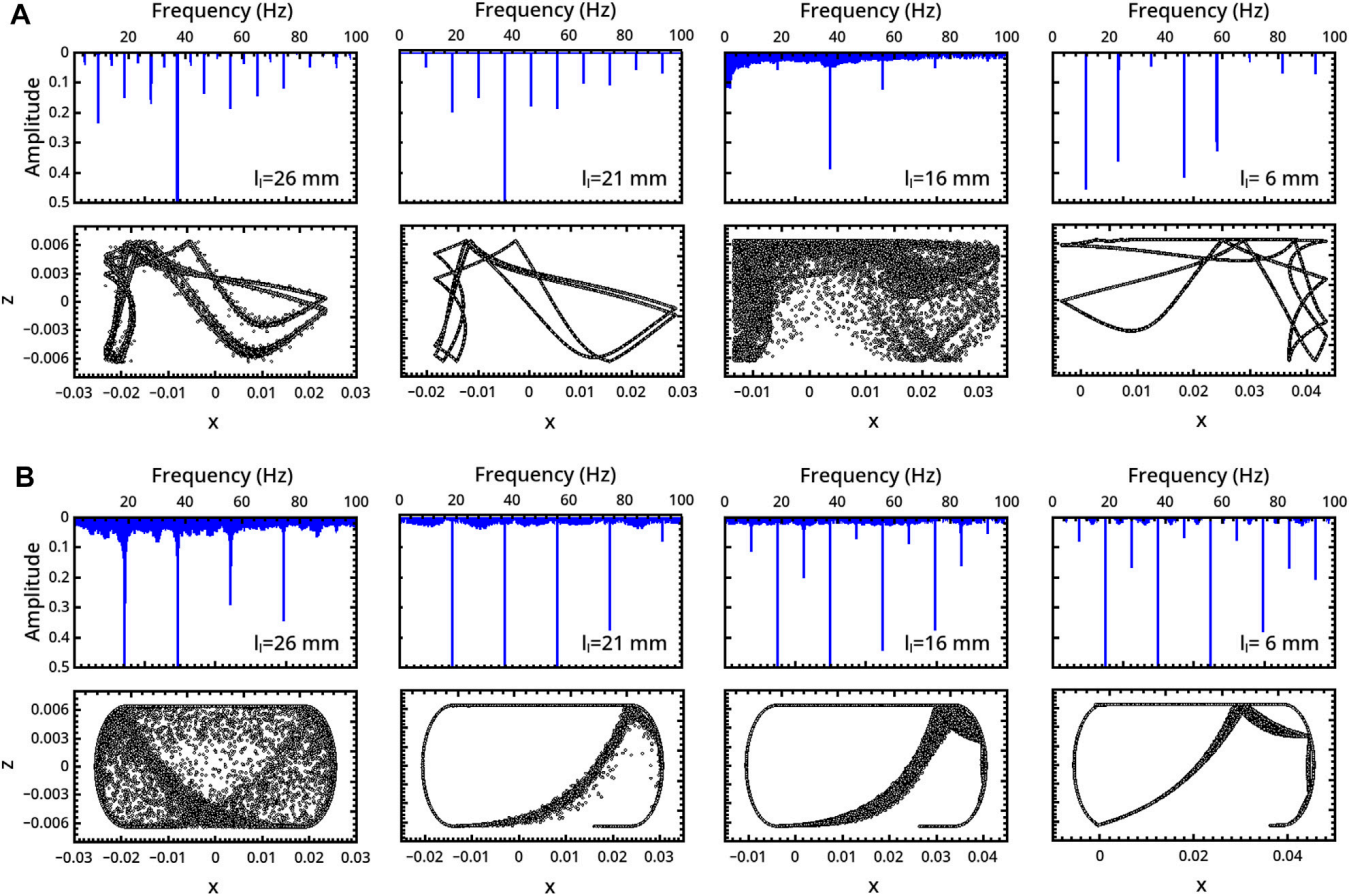
Characterization of the milling body dynamics in **(A)** flat- and **(B)** curved-base geometries as a function of the mechanical arm location, *l*_*l*_. For each regime, we report the trajectory (bottom) and the FFT of the time series describing the vertical displacement of the milling disc (top).

We finally explored the impact of an asymmetric forcing imposed on the disc when the mechanical arm is placed at different distances from the reactor center, for instance, decreasing *l*_*l*_ in favor of *l*_*r*_. The results for the two reactor geometries under consideration are summarized in Figure 10, where we report different dynamics observed by progressively decreasing *l*_*l*_ (increasing *l*_*r*_) by steps of 5 mm from the centered allocation, which is *l*_*l*_ = 26 mm (the case *f* = 0.5 and *r*_*d*_ = 3 mm is considered).

In the flat-base system (Figure 10A), the system firstly undergoes a transition from a P8 (*l*_*l*_ = 26 mm) to a P4 (*l*_*l*_ = 21 mm) periodicity, and then a chaotic regime for *l*_*l*_ = 16 mm occurs. Pushing further the geometrical asymmetry (*l*_*l*_ = 6 mm) the dynamics recovers a P1 behavior characterized by a main frequency which is half of the device forcing frequency.

In reactors with curved bases (Figure 10B), the asymmetry shows a “simplifying” effect in dynamical terms, inducing a transition from an initial chaotic regime for the centered arm to different kinds of periodicity: P1 (with the same devise working frequency) for *l*_*l*_ = 21 mm, P2 for *l*_*l*_ = 16 mm and P4 for *l*_*l*_ = 6 mm.

**FIGURE 11:**
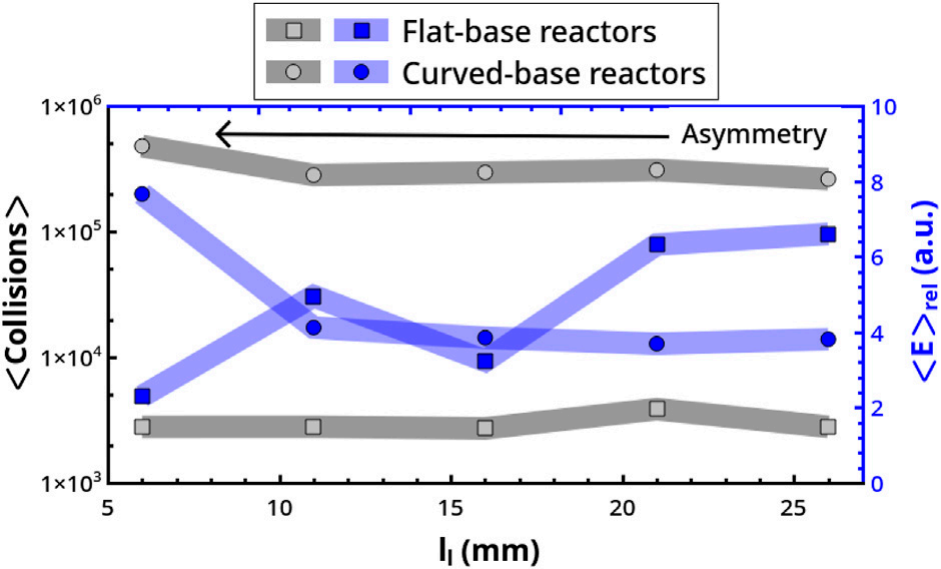
Dependence of ⟨Collisions⟩ and ⟨*E*⟩_*rel*_ on the forcing asymmetry as controlled by the mechanical arm location, *l*_*l*_.

As shown in Figure 11, in both geometries the average number of collisions on the bases is not substantially affected by the asymmetric position of the mechanical arm. However, the two reactor types follow opposite trends in energetic terms, with the average relative energy of the collisions decreasing with the asymmetry of the forcing (i.e., decreasing *l*_*l*_) in flat-base reactors. The opposite is true in curved-base reactors. A correlation between the typology of the dynamical regimes and collision energy can hardly be established. We can just point out that chaotic regimes correspond to a decrement of ⟨*E*⟩_*rel*_.

## 4 Concluding Remarks

The geometry of the reactor used in ball-milling approaches can profoundly impact the outcome of a mechanical process by affecting the dynamics of the milling body, which rules the powder dragging, mixing, and concretely transfers the mechanical energy to induce physico-chemical transformations at collision with other milling bodies or with the reactor walls.

In this work we have focused our attention on the dynamics of one milling body driven by a harmonic pendular force within two different geometries, namely reactors with flat and curved bases, which are among the most common devices in use. By using a 2-dimensional description of these systems, we have probed the dynamical response of a milling body to the variation of relevant parameters such as the milling body size and the restitution coefficient. These parameters present experimental interest and turn out to critically control the transition from periodic to chaotic behaviors in both the geometries considered, following period-doubling-like routes. Parametric regions where the systems show chaotic behaviors and, hence, potentially more efficient mixing, are observed in curved-base reactors, where aperiodic dynamics dominate in the presence of nearly elastic conditions and are also found in inelastic conditions. Although the energetic profiles of the two geometries as a function of the control parameters considered are essentially overlapping, curved-base reactors are characterized by a larger number of collisions, which could translate into a higher probability of inducing a transformation. A direct correspondence between the dynamic type and the energetics of the system cannot be established. In both systems, the number of collisions is larger in periodic inelastic conditions as these favor the slipping of the milling body on the reactor walls.

A more general criterion for controlling the dynamics within the systems relies on a suitably small change in the relative size of the reactor and that of the milling body. Here we identified two possible ways to maintain a given dynamic when the milling body size is varied: *i*) scaling the reactor size, including the length of the mechanical arm, of the same factor of the milling body size variation; *ii*) augmenting the reactor width and height by the same increment (or decrement) of the milling body radius. In both cases, a desired regime can be modulated in intensity (i.e., the collision energy can be either increased or decreased) while maintaining unaltered the dynamical features.

An asymmetric force on the milling body, which can be imposed by changing the position of the mechanical arm with respect to the reactor center, can also play a critical role in controlling the transition from periodic to chaotic regimes.

## Data Availability

The raw data supporting the conclusion of this article will be made available by the authors without undue reservation.

## Footnotes

1
https://www.cost.eu/stories/mechanochemistry-pushes-more-sustainable-processes.

2 While the horizontal component of the disc displacement is essentially Imposed by the working frequency of the devise, the vertical Displacement confers emergent features to the global dynamics (see Figure 1 of the Supplementary Material).
