# Supplementary material for: Controlling Nonlinear Dynamics of Milling Bodies in Mechanochemical Devices Driven by Pendular Forcing
Source: Front Chem. 2022 Aug 5;10:915217. doi: 10.3389/fchem.2022.915217 (PMC9388739; doi:10.3389/fchem.2022.915217)
Supplement: Supplementary file 1 [file DataSheet1.PDF]

Controlling nonlinear dynamics of milling bodies in mechanochemical devices driven by pendular forcing

Supplementary material

A. Polo,[1] M. Carta,[2] F. Delogu,[2] M. Rustici,[1] and M. A. Budroni[1]

[1] *Dipartimento di Chimica e Farmacia, Università degli Studi di Sassari, Via Vienna 2, Sassari 07100, Italy.*

[2] *Dipartimento di Ingegneria Meccanica, Chimica, e dei Materiali, Università degli Studi di Cagliari, via Marengo 2, Cagliari 09123, Italy.*

(Dated: 6 April 2022)

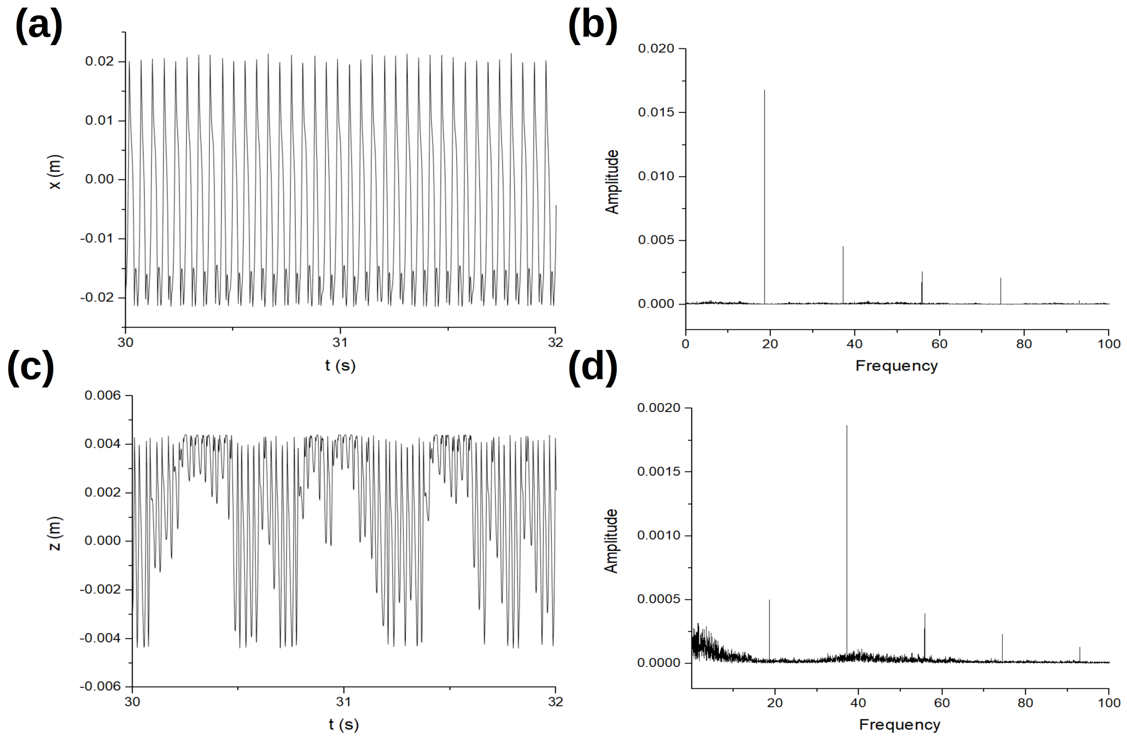

FIG. 1. Time series of the x- and z-displacement of the milling disc as a function of the time (f = 0.5, r_d = 3 mm). The oscillatory patterns and related fast Fourier transforms show that, while motion along the horizontal component essentially follows the forcing frequency, the vertical component presents higher complexity with the emergence of non-negligible short-frequency contributions.

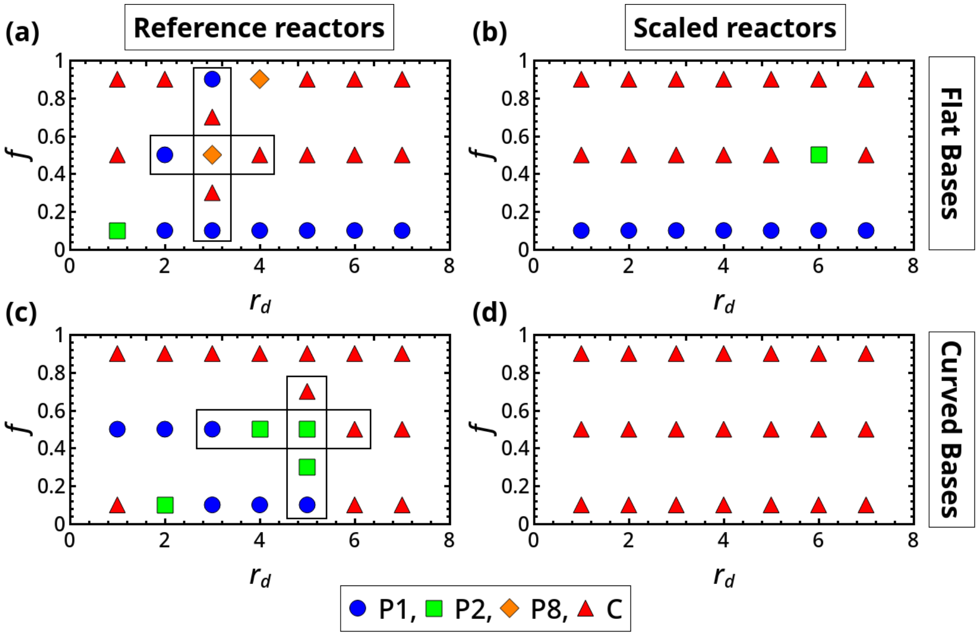

FIG. 2. Comparison between the main dynamical scenarios characterizing the milling body dynamics in the reference reactors and in those scaled according to case 1. The dynamics are classified in the parameter spaces spanning the restitution coefficient, f, and the disc radius, r_d. Panels (a, b) and (c,d) refer to flat- and curved-base reactors, respectively. P1, P2, P8 and C identify period-1, period-2, period-8 and chaotic regimes, respectively. The parallel between the reference and the scaled reactors show a general mismatch between the dynamics.

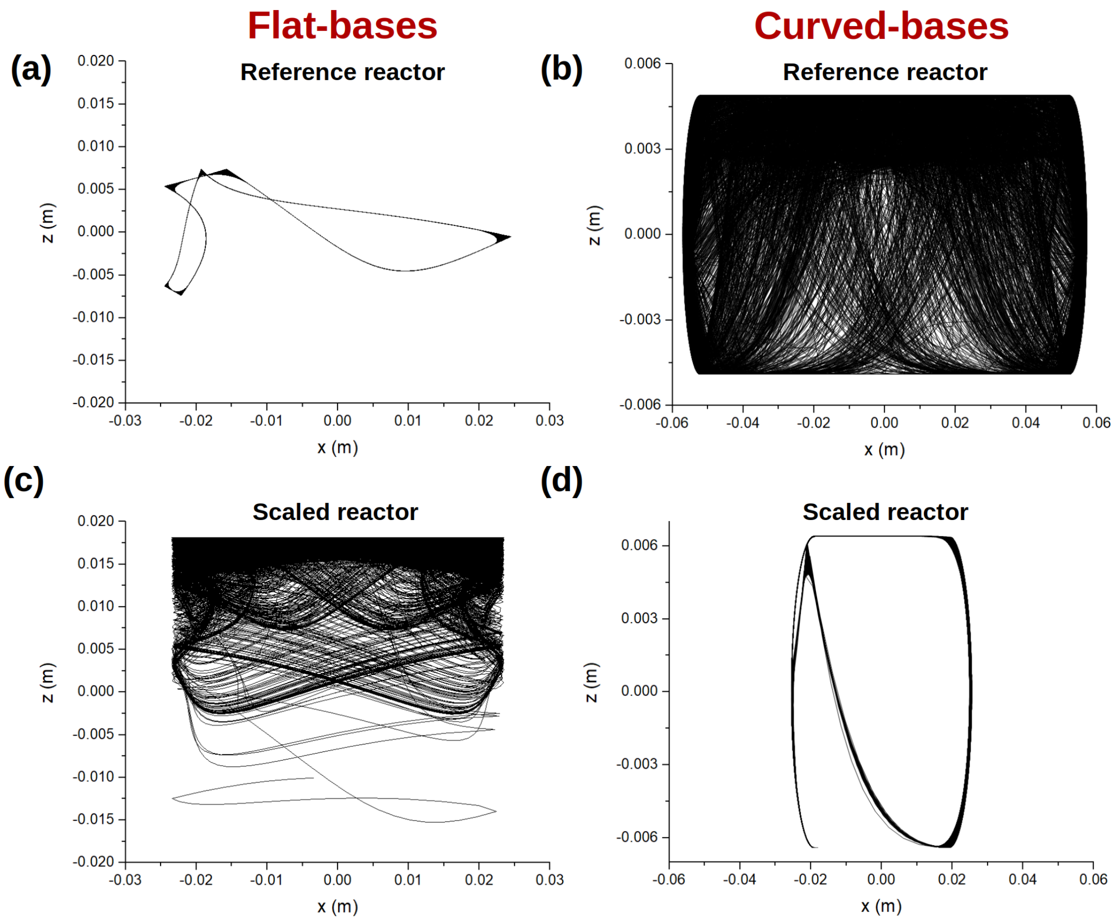

FIG. 3. Trajectories of the milling disc in reference reactors and in spatial domains scaled as described for case 1 (i.e. changing the width and the height of the reactor without preserving the aspect ratio, with the length of the mechanical arm, R, fixed) ($f = 0.5$). The reference systems are characterized by $r_d^1 = 3$ mm, the spatial domains are $\Omega_f^1 = 53$ mm x 18.8 mm for the flat-base reactors and Ω_c^1 =38.2 mm x 18.8 mm + π (9.4 mm)^2 for the curved-base reactors. The scaled reactors present $r_d^2 = 1.5 r_d^1$ =4.5 mm, $\Omega_f^2 = \Omega_c^2 = 2.25\Omega_f^1 = 2.25\Omega_c^1$ ($\Omega_f^2 = 53$ mm x 42.3 mm $\Omega_c^2 =$ 104.4 mm x 18.8 mm + π(9.4 mm)^2). The characterization of these dynamics are reported in Fig. 7a and 7b of the manuscript.

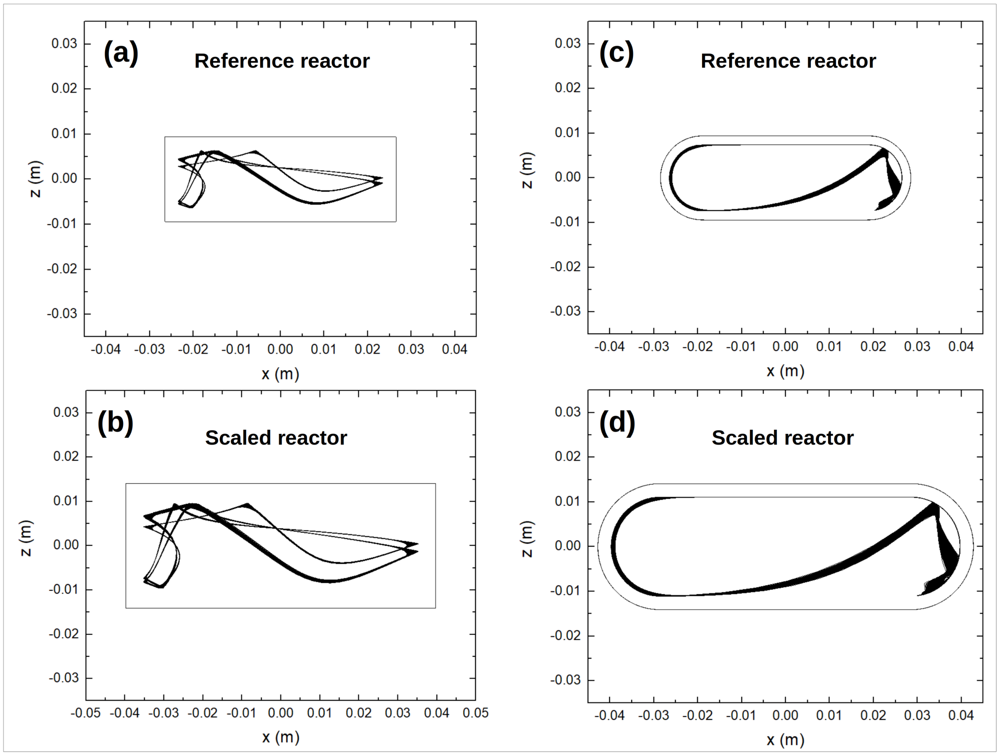

FIG. 4. Comparison between the trajectories followed by the milling disc in the reference spatial domain and in a reactor scaled following case 3 (i.e. scaling the whole system proportionally, including the length of the mechanical arm). The reference systems are characterized by $r_d^1 = 3$ mm, the spatial domains are $\Omega_f^1 = 53$ mm x 18.8 mm for the flat-base reactors and Ω_c^1 =38.2 mm x 18.8 mm + π (9.4 mm)^2 for the curved-base reactors. The scaled reactors present $r_d^2 = 1.5r_d^1$ =4.5 mm, Ω_f^2 = 78.45 mm x 28.2 mm; Ω_c^2 =57.3 mm x 28.2 mm + π(14.1 mm)^2; R = 183 mm. The coordinates of the trajectory appear scaled of the same factor scaling the reactor size (x 1.5). The characterization of these dynamics is given in Fig. 7c and 7d.

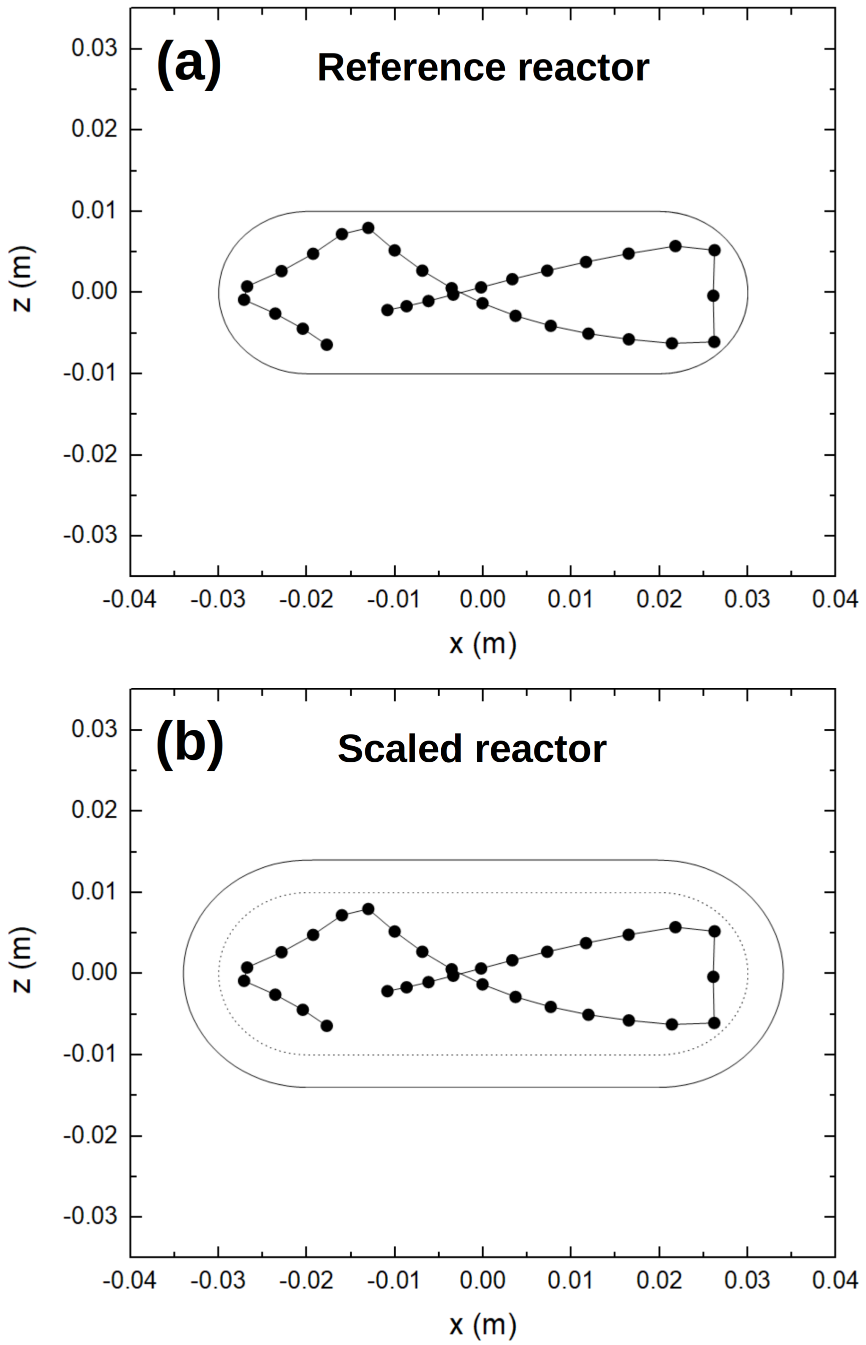

FIG. 5. Exemplar comparison between segments of the trajectory followed by the milling disc in the reference domain and that adapted according to relations 7-9 in a curved-base reactor.
